# Inhibitory effects of acetyl-11-keto-β-boswellic acid (AKBA) on human cytomegalovirus (HCMV) in vitro

**DOI:** 10.71150/jm.2601007

**Published:** 2026-03-25

**Authors:** Bingquan Chu, Zhiwei Ding, Xinna Wu, Yunchuang Chang, Chunxia Wu, Yicheng Fu, Genxiang Mao, Sanying Wang

**Affiliations:** 1 School of Biological and Chemical Engineering, Zhejiang University of Science and Technology, Hangzhou 310023, P. R. China; 2 Zhejiang Key Laboratory of Geriatrics and Geriatrics Institute of Zhejiang Province, Zhejiang Hospital, Hangzhou 310030, P. R. China

**Keywords:** antiviral, acetyl-11-keto-β-boswellic acid, human cytomegalovirus, NR4A1

## Abstract

This study presents the first investigation of acetyl-11-keto-β-boswellic acid (AKBA)’s anti-human cytomegalovirus (HCMV) activity in vitro and elucidates its underlying mechanisms. In HCMV Towne strain-infected WI-38 cells, AKBA (1-12 μM) exhibited negligible cytotoxicity while significantly suppressing virus-induced cytopathic effects (CPE) at 6–10 μM, with dose-dependent reduction of viral proteins (IE1/2 and p52) expression, viral DNA copy number (*UL123*, *UL44*, and *UL32*), and infectious viral progeny titer (TCID_50_). Time-of-addition experiments demonstrated the primary antiviral activity of AKBA during post-entry phase, along with direct virion inactivation. Transcriptome analysis revealed that AKBA significantly downregulated the expression of the host factor NR4A1 induced by HCMV, a finding further validated by Western blotting. Further gene knockdown experiments confirmed that silencing NR4A1 significantly reduced the expression of viral proteins IE1/2, thereby validating NR4A1 as a key host factor for HCMV infection. These findings indicate that AKBA has a potent and dose-dependent inhibitory effect on HCMV replication in WI-38 cells, and proves that this effect is mediated through two different mechanisms: one is the downregulation of the expression of the key host factor NR4A1, and the other is the direct inactivation of HCMV viral particles.

## Introduction

Human cytomegalovirus (HCMV), an enveloped betaherpesvirus, represents a globally ubiquitous pathogen demonstrating a seropositivity rate exceeding 90% seroprevalence in the humans (Zuhair et al., 2019). Its characteristic pathogenic mechanism involves establishing lifelong latent infection, with immunosuppression triggering rapid reactivation and recurrent infections (Crough and Khanna, 2009; Griffiths and Reeves, 2021). Clinically, HCMV is an established etiological factor in pregnancy loss and congenital defects, manifesting as microcephaly, sensorineural deafness, and neurocognitive impairment (Britt, 2018; Messinger et al., 2020).

The HCMV genome consists of double-stranded DNA (dsDNA) with a size of 236 kb, constituting the largest known human herpesvirus genome (Li et al., 2021; Van Damme and Van Loock, 2014). Following entry into host cells, nucleocapsids undergo microtubule-mediated nuclear translocation for DNA release and replication, expressing over 165 viral proteins (Kalejta, 2008). HCMV gene expression follows a temporal cascade: Immediate Early (IE), Early (E), and Late (L) phases (Isomura and Stinski, 2013; Rozman et al., 2022). The IE genes “*UL123*/*UL122*” (encoding IE1/IE2 proteins) undergo expression during initial infection and are essential for initiating subsequent viral gene transcription (García-Ramírez et al., 2001). E genes, expressed prior to viral DNA replication, encode proteins mediating viral genome replication and host cell functional modulation. L genes, expressed following the initiation of viral DNA replication, primarily encode structural components essential for virion assembly (Isomura and Stinski, 2013).

Despite substantial disease burden, no licensed HCMV vaccine has been approved for clinical use (Gourin et al., 2023; Rand et al., 2020). Current therapeutics including ganciclovir, foscamet sodium (FOS or PFA), and cidofovir inhibit viral DNA polymerase but incur dose-limiting toxicities (myelosuppression, nephrotoxicity, and electrolyte imbalances) and face drug-resistant challenges (Atanasoff et al., 2025; Chen et al., 2022). Consequently, developing novel anti-HCMV agents with high efficacy and low toxicity is urgently needed (Wild et al., 2023).

**Fig. 1.**
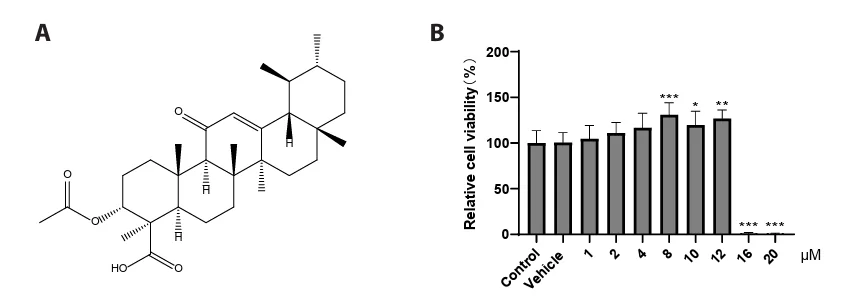
(A) Chemical structural formula of AKBA. (B) Effects of AKBA on HCMV host cells - human embryonic lung fibroblast cells (WI-38) cytotoxicity as determined by CCK8 assay. The vehicle (DMSO, v/v = 0.05%) treatment showed no significant difference compared to the control group. *p < 0.05, **p < 0.01, ***p < 0.001 vs. Control group.

Frankincense, an oleo-gum resin secreted by *Boswellia* spp. (Burseraceae family), contains bioactive terpenoids and volatile oils (Gong et al., 2022; Kosolapov et al., 2025). Acetyl-11-keto-β-boswellic acid (AKBA), its principal pentacyclic triterpenic acid (Fig. 1A), exerts anti-inflammatory, antioxidant, cardioprotective activities (Lauß et al., 2024), clinically applied in nonspecific inflammatory diseases (Han et al., 2025; Nischang et al., 2023).

Our preliminary screening results indicated that AKBA exhibited significant anti-HCMV activity. Thus, the present study aimed to evaluate the effects of AKBA on the viability of WI-38 cells, establish its dose-response efficacy against HCMV, and identify the critical stages of the viral lifecycle targeted by AKBA. Furthermore, we investigated the key mediators in HCMV-infected WI-38 cells treated with AKBA through integrated transcriptomics and Western blotting, followed by functional validation using siRNA-mediated gene knockdown. These findings contribute to understanding the anti-HCMV activity and mechanism of action of AKBA, and its potential as an antiviral candidate merits further research.

## Materials and Methods

### Materials, virus, and cells

Human diploid fibroblast cells (WI-38) and HCMV (Towne strain) were obtained from the American Type Culture Collection (USA). Dulbecco’s modified Eagle’s medium (DMEM, high glucose), fetal bovine serum (FBS), penicillin-streptomycin solution (100×), and trypsin-EDTA (0.25%) were purchased from Gibco-BRL Life Technologies (USA). Acetyl-11-keto-β-boswellic acid (AKBA) was procured from Shanghai Standard Technology Co., Ltd. (China). The positive control drug Foscamet sodium (FOS) was acquired from Santa Cruz Biotechnology (USA). Cell lysis buffer for Western blotting, SDS-PAGE protein sample loading buffer (5×), Universal SYBR Green Fast qPCR Mix (2×), and CCK-8 kit were all acquired from ABclonal Technology (China). Tris-glycine SDS-PAGE running buffer (10×) and Tris-glycine transfer buffer (10×) were purchased from Sangon Biotech (China). QIAamp DNA Mini Kit was acquired from Qiagen (Germany). Mouse monoclonal antibodies against HCMV immediate-early protein (IE 1/2) and early protein (p52) were sourced from Virusys Corporation (USA). NR4A1 protein antibody was purchased from Cell Signaling Technology (USA). GAPDH antibody was acquired from ABclonal Technology (China). Small interfering RNAs (siRNAs) targeting NR4A1 (siNR4A1-1, siNR4A1-2, and siNR4A1-3), non-targeting control siRNA (NC), GAPDH siRNA (siGAPDH), and all primers were purchased from Tsingke Biotechnology (China). The Starvio siRNA Transfection Reagent was purchased from Shanghai Starvio Biotechnology Company Limited (China).

### Cell culture, HCMV infection, and AKBA treatment

WI-38 cells were cultured in DMEM containing 10% fetal bovine serum (FBS) and 1% penicillin-streptomycin solution. HCMV Towne strain was prepared according to established protocols (Mao et al., 2016; Wang et al., 2020). For G_0_ phase synchronization, cells were subjected to DMEM with 0.2% FBS 48 h prior to infection. Two h before HCMV inoculation, medium was replaced with drug-containing solutions (FOS or AKBA). After 2 h, cells were infected with HCMV at a multiplicity of infection (MOI) of 0.5. Cell samples were collected at predetermined time points for subsequent preparation of protein and viral DNA extracts to evaluate antiviral effects. In all experiments conducted in this study, the “Mock” group refers to normal WI-38 cells without any drug treatment or viral infection. The “HCMV only” group represented cells infected with HCMV alone without any drug intervention. AKBA was prepared as a 40 mM stock solution in dimethyl sulfoxide (DMSO). The vehicle used was dimethyl sulfoxide (DMSO). In each experiment, the vehicle control groups received an equivalent volume of DMSO corresponding to that present in the highest tested concentration of AKBA.

### CCK-8 cell viability assay

The impact of AKBA on WI-38 cell viability was assessed using the Cell Counting Kit-8 (CCK-8). WI-38 cells were seeded in 96-well plates (5 × 10^3^ cells/well) and cultured under standard conditions (37°C, 5% CO_2_) for 24 h. Medium was then replaced with fresh DMEM containing serially diluted AKBA (1, 2, 4, 8, 10, 12, 16, and 20 μM) supplemented with 0.2% FBS and 1% penicillin-streptomycin for 5 days. Subsequently, 10 μl CCK-8 solution was added per well, mixed by gentle shaking, and incubated at 37°C for 1–2 h. Absorbance was measured at 450 nm using a Thermo Scientific Multiskan^TM^ FC microplate reader. Relative cell viability was normalized to untreated controls (100%). Experiments were independently repeated three times (n = 3).

### Time‑of‑addition experiments

G_0_-synchronized WI-38 cells were infected with HCMV (MOI = 0.5) for 2 h. AKBA (8 µM) or 0.02% DMSO (v/v) was administered at specified time points. Treatments were terminated by medium removal, and wash cells three times with PBS, and fresh medium replenishment. Cell samples were collected at 96 h post-infection (hpi) for Western blot analysis. Six different treatment protocols were implemented as follows:

**Group 1 (Full time treatment, Full time-T):** AKBA was added 2 h prior to HCMV inoculation; following 2 h of HCMV infection, the medium containing AKBA and HCMV was discarded. Cells were washed with PBS and replenished with fresh DMEM containing 0.2% FBS and AKBA.

**Group 2 (Pre-treatment cell, Pre cell-T):** Cells were pre-treated with AKBA for 2 h before HCMV inoculation; culture medium was removed and cells were washed with PBS. Then fresh DMEM with 0.2% FBS was added, followed by immediate HCMV inoculation. After 2 h post-inoculation, the HCMV-containing medium was discarded, cells were washed with PBS, and then replaced with fresh DMEM with 0.2% FBS.

**Group 3 (Co-treatment, Co-T):** AKBA and HCMV were added concurrently; after 2 h, the medium containing AKBA and HCMV was discarded. Cells were washed with PBS and replenished with fresh DMEM with 0.2% FBS.

**Group 4 (Pre-treatment virus and drug, Pre virus & drug-T):** AKBA and HCMV were mixed and incubated at 37°C for 2 h; the AKBA-HCMV mixture was subsequently added to the cells. After 2 h, the medium containing AKBA and HCMV was discarded. Cells were washed with PBS and replaced with fresh DMEM with 0.2% FBS.

**Group 5 (Post-treatment, Post-T):** After HCMV inoculation alone for 2 h, the HCMV-containing medium was removed, washed three times with PBS and replacement with fresh DMEM containing 0.2% FBS and AKBA.

**Group 6 (Pre-treatment virus, Pre virus-T):** HCMV was mixed with DMEM containing 0.2% FBS and incubated at 37°C for 2 h; the HCMV-medium mixture was then added to the wells simultaneously with AKBA. After 2 h, the medium containing HCMV and AKBA was discarded. Cells were washed with PBS and replaced with fresh DMEM with 0.2% FBS.

### Western blotting

Cells were harvested by scraping and lysed with RIPA buffer for total protein extraction. Protein concentration was quantified using the BCA assay, followed by loading buffer preparation. Protein samples (50 μg) were separated by SDS-PAGE and electrotransferred onto PVDF membranes. Membranes were blocked with 5% skim milk at room temperature for 1 h, then incubated overnight at 4°C with primary antibodies. After washing, membranes were incubated with secondary antibodies at 37°C for 1 h. Target bands were visualized using enhanced chemiluminescence (ECL). HCMV-infected and FOS-treated groups served as experimental controls.

### qPCR

Cell samples were prepared as described above. Culture supernatants were discarded, and cells were washed with PBS before trypsinization (0.25% trypsin-EDTA). HCMV DNA was extracted using the QIAamp DNA Mini Kit according to the manufacturer’s instructions. HCMV DNA copy numbers were quantified using 2× Universal SYBR Green Fast qPCR Mix with primers targeting *UL123*, *UL44*, *UL32*, and *GAPDH* (Table 1). The thermal cycling protocol was performed as follows: initial denaturation (95°C, 3 min); 40 cycles of denaturation (95°C, 5 s) and annealing/extension (60°C, 30 s). Technical triplicates (n = 3) were performed for each sample. The difference in HCMV DNA copy numbers between AKBA-treated groups and HCMV-infected alone group was calculated using the 2^−ΔΔCt^ method. First, the internal reference gene *GAPDH* was used to normalize the Ct values of all treated and control samples (ΔCt = Ct (sample) - Ct (*GAPDH*)). Second, the Ct values of AKBA-treated groups were compared with those of control samples. ΔΔCt = ΔCt (sample) - ΔCt (control sample). The relative expression of target genes was expressed as 2^-ΔΔCt^.

### TCID_50_

The TCID_50_ assay was performed in 96-well plates. When WI-38 cells reached approximately 80% confluence, the viral stock was serially diluted 10-fold. From each dilution, 100 µl was inoculated onto WI-38 cells and cultured at 37°C under 5% CO_2_. Each dilution was tested in eight replicate wells. The appearance of cytopathic effects (CPE) was monitored and recorded daily under a microscope until the number of CPE-positive wells stabilized. Finally, the 50% tissue culture infectious dose (TCID_50_) was calculated according to the Reed–Muench method.

### Transcriptome RNA sequencing analysis

WI-38 cells infected with HCMV (MOI = 0.5) were divided into four groups: DMSO + Mock, DMSO + HCMV, AKBA + HCMV, and AKBA + HCMV, with three biological replicates per group. Cells were collected at 3 days post-infection (3 dpi), and total RNA was extracted using TRIzol reagent (Invitrogen). Sequencing was performed by OmicStudio (LC-Bio Technology Co., Ltd., China). Strict quality control was performed using an Agilent 2100 Bioanalyzer with RNA 6000 Nano Kit (RIN > 7.0), followed by quantification using a NanoDrop ND-1000 spectrophotometer (concentration > 50 ng/μl, total amount > 1 μg). For library preparation, polyadenylated [poly(A)] mRNA was enriched through two rounds of oligo(dT) magnetic bead selection (Thermo Fisher). mRNA was fragmented at 94°C for 6 min using the NEBNext Magnesium RNA Fragmentation Module, then reverse-transcribed to cDNA using SuperScript II Reverse Transcriptase. During second-strand synthesis, double-stranded cDNA was constructed by incorporating dUTP (Thermo Fisher) with *E. coli* DNA Polymerase I (NEB) and RNase H (NEB). After end repair and adapter ligation, strand-specific libraries were prepared via UDG enzyme (NEB) digestion. Final libraries (300 ± 50 bp) were amplified through 8 PCR cycles (98°C/15 s, 60°C/15 s, 72°C/30 s). Sequencing was performed on the Illumina NovaSeq 6000 platform (LC-Bio) in 150 bp paired-end mode (PE150). Raw data were processed by fastp to remove adapter sequences and low-quality bases, followed by alignment to the human reference genome GRCh38 using HISAT2. Transcript assembly and FPKM quantification were conducted with StringTie. Differentially expressed genes (DEGs) were identified by DESeq2 analysis (|log2 fold change| > 1, adjusted P < 0.05). All sequencing data were deposited in the GEO database (accession no. GSE289362). Differential expression gene (DEG) analysis was conducted using the OmicStudio cloud platform (https://www.omicstudio.cn).

### siRNA transfection and knockdown experiment

WI-38 cells were seeded in 12-well plates, with 1 ml of DMEM with 10% FBS added per well, and cultured overnight at 37°C under 5% CO_2_. Transfection was performed when cell density reached 30–50%. Before transfection, 3 μl of Starvio transfection reagent was mixed with 100 μl of Trans Enhancer reagent and incubated at room temperature for 5 min. Then, 24 pmol of siRNA (Table 2) was added, gently mixed, and incubated at room temperature for 15 min to form the transfection complex. 100 μl of the complex was added to each well, and the culture plate was gently shaken to mix. After transfection, the cells continued to be cultured in a 37°C incubator for 48 h. Subsequently, the medium was aspirated and replaced with DMEM with 0.2% FBS. After the medium change, HCMV Towne strain (MOI = 0.5) was immediately inoculated. Cells were harvested 72 h post-infection for total protein extraction, used for subsequent Western blot analysis. In the experiment, non-targeting siRNA (NC) was set as a negative control, and GAPDH siRNA (siGAPDH) was used as a positive control for transfection efficiency.

### Statistical analysis

The half-maximal cytotoxic concentration (CC_50_) and half-maximal inhibitory concentration (IC_50_) were calculated by fitting the dose-response data (obtained from CCK-8 and TCID_50_ assays, respectively) to a nonlinear regression model using GraphPad Prism software (version 9.5.1). Data are presented as Mean ± standard deviation (SD). *p*-Values calculated by one-way ANOVA. Statistical significance was defined at *p* < 0.05.

## Results

### Effects of AKBA on WI-38 cell viability

To evaluate the potential cytotoxicity of AKBA against HCMV host cells (WI-38) and to determine its safe concentration range. The cytotoxicity of AKBA in WI-38 was assessed using CCK-8 assay (Fig. 1B). Following 5 days of treatment, AKBA concentrations ranging from 1 to 12 μM exhibited no significant cytotoxicity toward WI-38 cells, whereas pronounced cytotoxic effects were observed at 16 μM and 20 μM (CC_50_ = 15.33 μM). These results demonstrate that AKBA is non-cytotoxic to HCMV host cells in vitro within the 1–12 μM concentration range.

### Anti-human cytomegalovirus (HCMV) activity of AKBA

**Fig. 2.**
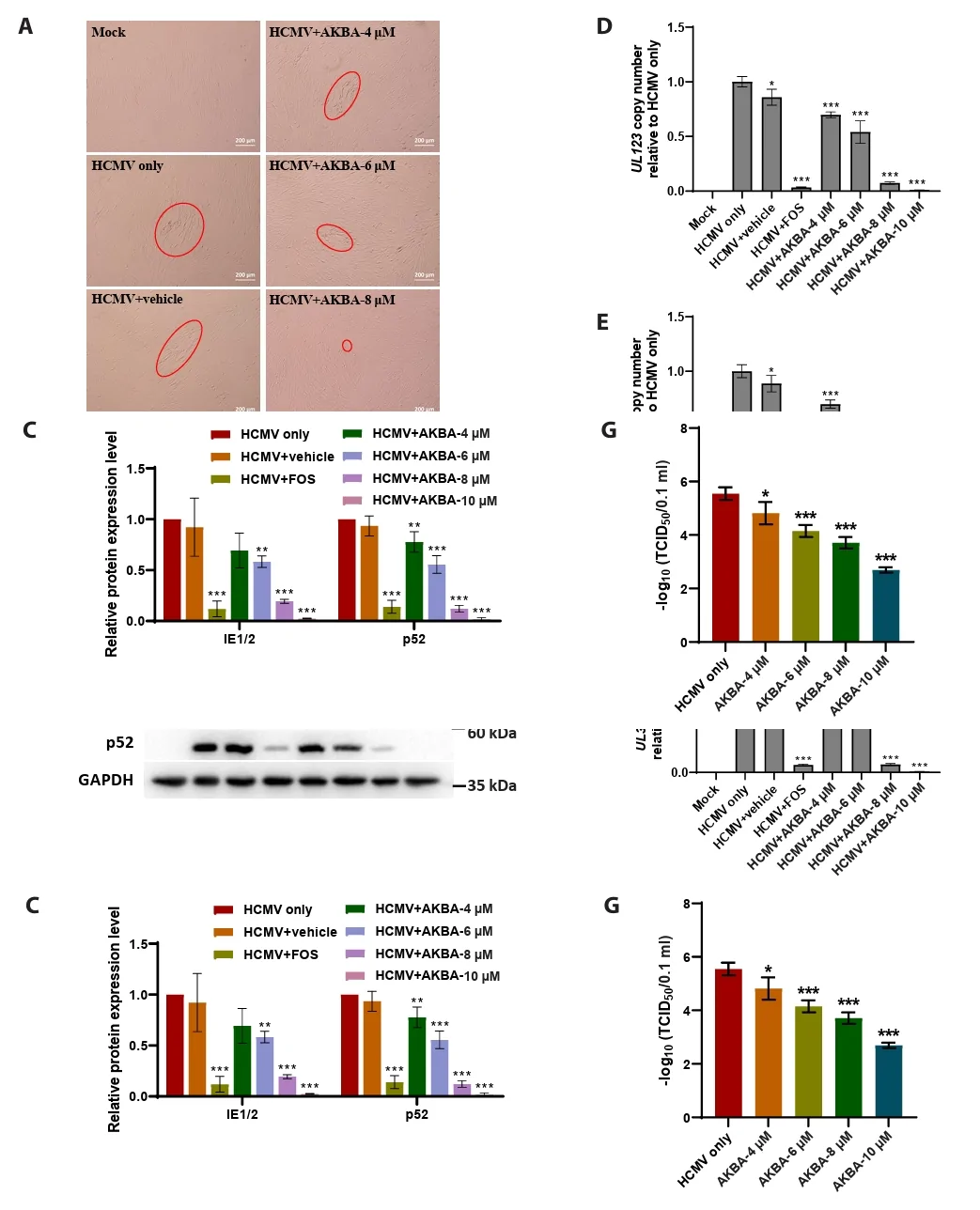
Anti-HCMV activity of AKBA in WI-38 cells. (A) Effect of AKBA on HCMV-induced cytopathic effects (CPE). WI-38 cells infected with HCMV Towne strain (MOI = 0.5) were pretreated with indicated concentrations of AKBA (4, 6, 8, and 10 μM) 2 h prior to inoculation. Cellular morphology was assessed at 5 days post-infection (dpi). Representative CPE are indicated by red circles. Images shown are representative of three independent experiments. (B) Expression levels of HCMV proteins IE1/2 and p52 in AKBA-treated WI-38 cells. (C) Quantitative analysis of protein expression. Cells were pretreated with AKBA (4–10 μM) 2 h before HCMV infection (MOI = 0.5). Total protein was extracted at 5 dpi for Western blotting analysis. GAPDH served as a loading control. Protein band intensities were quantified using ImageJ (n = 3). (D–F) qPCR analysis of HCMV DNA copy numbers: (D) immediate-early gene *UL123*, (E) early gene *UL44*, and (F) Late gene *UL32*. All HCMV-infected groups (MOI = 0.5) were harvested at 5 dpi for DNA extraction. (G) Inhibition of infectious viral progeny production by AKBA. The infectious viral titer in the supernatant of HCMV-infected WI-38 cells was determined by TCID_50_ assay at 5 dpi. The viral titer is expressed as Log_10_ TCID_50_/0.1 ml. AKBA-treated groups (4–10 μM) were compared with HCMV only group. FOS was used as a positive control for anti-HCMV activity. The vehicle control contained 0.025% (v/v) DMSO. *p < 0.05, **p < 0.01, ***p < 0.001 vs. HCMV only group.

To evaluate the effect of AKBA on HCMV-induced cytopathic effect (CPE), we observed the CPE produced by HCMV-infected host cells and the morphological changes in infected groups treated with different concentrations of AKBA (Fig. 2A). WI-38 cells infected with HCMV at MOI = 0.5 exhibited significant morphological changes and CPE formation at 5 days post-infection (dpi). AKBA at 6 μM, 8 μM, and 10 μM significantly reduced HCMV-induced CPE (indicated by red circles in Fig. 2A). Notably, cells pre-treated with 10 μM AKBA showed no CPE, similar to the Mock group. These findings indicate that AKBA effectively protects WI-38 cells from HCMV-induced morphological damage.

To verify the anti-HCMV activity of AKBA at the protein level, we examined viral protein expression in the presence or absence of AKBA using Western blot analysis (Fig. 2B and 2C). AKBA significantly inhibited the expression of both HCMV immediate-early proteins (IE1/2) and early protein p52 in WI-38 cells. Relative to the HCMV only group: 4 μM AKBA reduced IE1/2 and p52 expression to 69.3% (*p* = 0.0539) and 77.75% (**p < 0.01), respectively; 6 μM reduced expression to 58.32% (**p < 0.01) and 55.55% (***p < 0.001); 8 μM reduced expression to 19.36% (***p < 0.001) and 12.2% (***p < 0.001); 10 μM reduced expression to 2.46% (***p < 0.001) and 1.72% (***p < 0.001).

To further confirm whether AKBA’s anti-HCMV activity at the genetic level matched its protein level effects, we measured viral DNA copy numbers in the presence or absence of AKBA treatment using qPCR (Fig. 2D, 2E, and 2F). Results showed that AKBA at the concentrations of 4, 6, 8, and 10 μM significantly inhibited the DNA copy numbers of HCMV genes (*UL123*, *UL44*, and *UL32*). Compared to the HCMV only group, 4 μM AKBA reduced *UL123*, *UL44*, and *UL32* levels to approximately 70% (***p < 0.001), while 6 μM AKBA further decreased them to around 50–54% (***p < 0.001). Notably, 8 μM AKBA strongly suppressed these levels to 5–7% (***p < 0.001), and 10 μM AKBA nearly abolished viral DNA copy numbers (< 1%; ***p < 0.001). These qPCR findings closely aligned with the Western blot data, collectively confirming AKBA’s potent inhibitory effect against HCMV infection in WI-38 host cells.

To directly assess the impact of AKBA on the production of infectious viral progeny, we determined the viral titer in the supernatant of infected cells using the TCID_50_ assay (Fig. 2G). The results showed that AKBA significantly and dose-dependently reduced the yield of infectious viral particles. Compared to the HCMV only group, treatment with 4, 6, 8, and 10 μM AKBA reduced the viral titer (Log_10_ TCID_50_/0.1 ml) from 5.55 ± 0.15 in the control group to 4.82 ± 0.43 (*p < 0.05), 4.15 ± 0.23 (***p < 0.001), 3.71 ± 0.21 (***p < 0.001), and 2.69 ± 0.10 (***p < 0.001), respectively. Based on the dose-response curve, the IC_50_ of AKBA against HCMV replication was calculated to be 3.04 μM. This indicates that AKBA not only inhibits viral protein expression and DNA copy numbers but also effectively blocks the production of infectious mature viral particles.

### AKBA exhibits antiviral effects during post-entry phase of HCMV infection and in vitro conditions

**Fig. 3.**
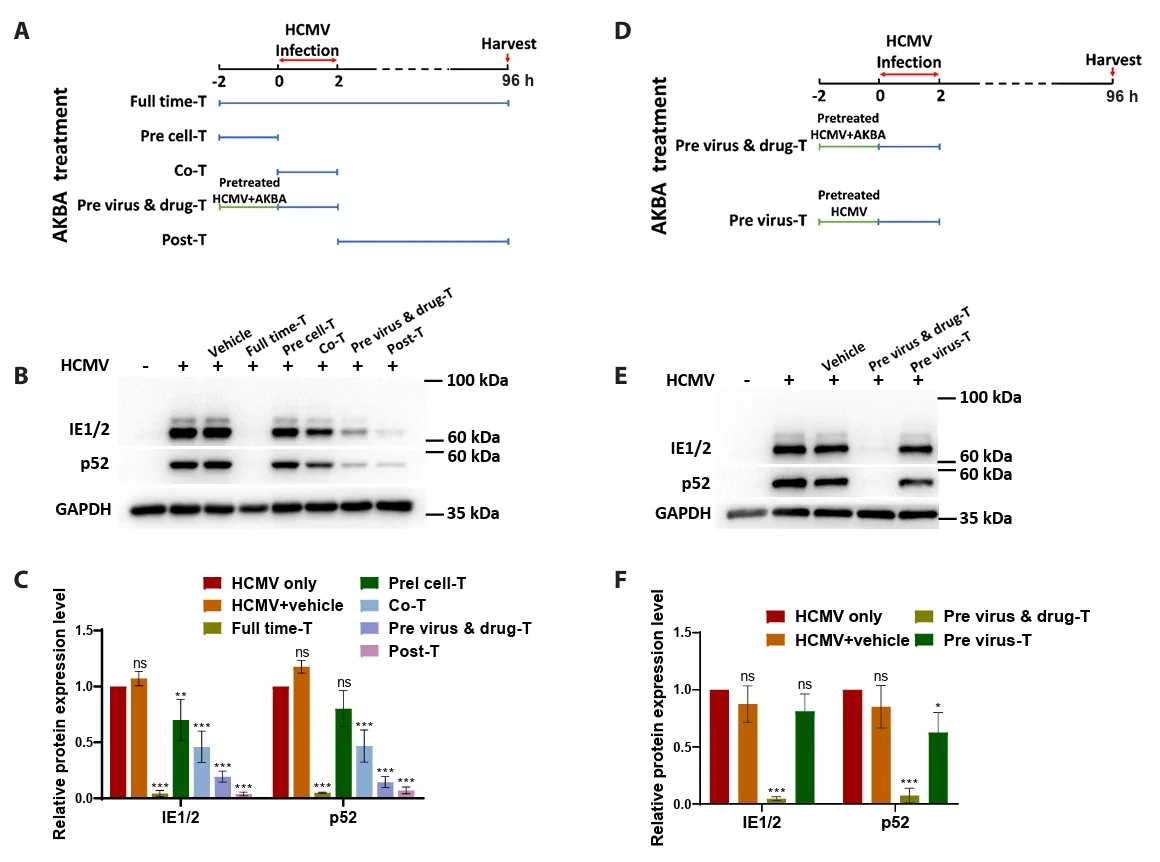
Stage-specific antiviral activity of AKBA against HCMV. (A, D) Schematic diagrams of experimental timelines for drug and virus addition. Black lines: Experimental timeline; red lines: Period of virus presence; blue lines: Period of drug treatment; green lines: In vitro pre-incubation of virus at 37°C. Full time-T: AKBA was present in the culture medium 2 h prior to infection, throughout the 2-h infection period, and was maintained thereafter until sample collection. Pre cell-T: Cells were pre-treated with AKBA for 2 h before infection; the drug-containing medium was removed immediately prior to viral inoculation. Co-T: AKBA and HCMV were added to the cells simultaneously at the onset of the infection period. Pre virus & drug-T: HCMV pre-incubated with AKBA in vitro before infection. Post-T: AKBA was added after HCMV infection. Pre virus-T: HCMV was pre-incubated in medium alone before infection. (B, E) Western blot analysis of protein expression in WI-38 cells infected with HCMV (MOI = 0.5) and treated with AKBA (8 µM) at designated time points. Cells were harvested at 96 h post-infection (hpi). GAPDH served as a loading control. (C, F) Quantitative analysis of protein expression levels from three independent experiments. Statistical significance was determined relative to the HCMV only group. The vehicle control contained 0.02% (v/v) DMSO. *p < 0.05, **p < 0.01, ***p < 0.001; ns, not significant vs. HCMV only group.

To clarify the stage at which AKBA inhibits HCMV infection, we performed time-of-addition experiments. Cells were treated and infected according to schematics in Fig. 3A and 3D. Viral protein (IE1/2 and p52, Fig. 3B, 3C, 3E, and 3F) expression was analyzed at 96 h post-infection (hpi). Compared with the HCMV only group, Group 1 (Full time-T) showed significantly reduced viral protein expression, confirming AKBA’s anti-HCMV activity (Fig. 3B and 3C). Compared analysis of Group 2 (Pre cell-T) versus HCMV only controls revealed that Group 2 (Pre cell-T) showed no significant p52 reduction but decreased IE1/2 levels, indicating AKBA pretreatment did not primarily target the viral replication stage (Fig. 3B and 3C). Group 3 (Co-T) also showed significant viral protein reduction, suggesting early intervention during HCMV infection (Fig. 3B and 3C). Conversely, Group 5 (Post-T) exhibited marked reduction in both IE1/2 and p52 (***p < 0.001), identifying the post-entry phase as AKBA’s principal antiviral stage (Fig. 3B and 3C).

Notably, compared to Group 3 (Co-T), Group 4 (Pre virus & drug-T) exhibited a more pronounced inhibition of viral proteins, suggesting that antiviral activity was already exerted during the “Pretreated HCMV + AKBA” phase (Fig. 3B and 3C). This implied that AKBA might directly inactivate HCMV during this phase. However, it remained unclear whether this effect was due to the natural decay of viral activity in vitro. To determine whether the in vitro condition itself was a major contributing factor to the reduction of HCMV proteins, we designed the experiment illustrated in Fig. 3D. The results showed that viral protein expression in the “Pre virus & drug-T” group was significantly lower than that in the “Pre virus-T” group (Fig. 3E and 3F). This indicated that “Pretreated HCMV” alone in the “Pre virus-T” group was not the primary cause of the eventual reduction in viral protein expression. Thus, short-term incubation in vitro had minimal impact on HCMV activity. Therefore, the observed inhibition should be primarily attributed to the presence of AKBA in the “Pretreated HCMV + AKBA” system. Collectively, these results confirm that AKBA can directly inactivate free HCMV particles.

In summary, time-of-addition experiments indicate that AKBA exerts its antiviral activity primarily during the post-entry replication phase of the virus, while also directly inactivating HCMV particles.

### Transcriptomic analysis reveals that AKBA suppresses HCMV-induced NR4A1 expression

To elucidate the responses of host WI-38 cells to HCMV infection and AKBA treatment, RNA sequencing was performed to analyze gene expression profiles across four experimental groups (DMSO + Mock, AKBA + Mock, and HCMV-infected groups treated with either DMSO or AKBA).

**Fig. 4.**
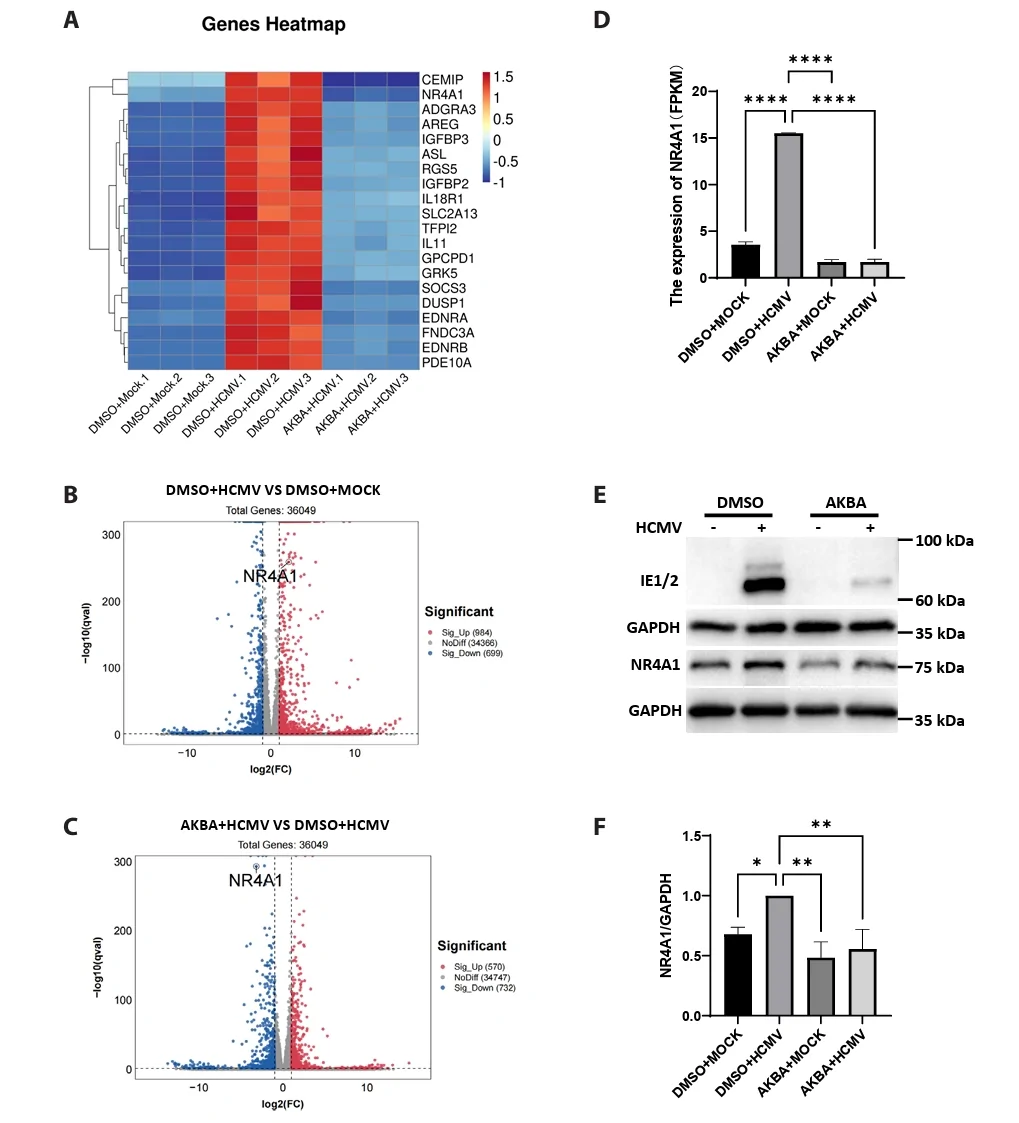
Effects of AKBA on HCMV-induced NR4A1 expression in WI-38 cells. (A) Heatmap analysis of differentially expressed genes (DEGs) in the DMSO + Mock, DMSO + HCMV, and AKBA + HCMV treatment groups. (B, C) Volcano plots of DEGs showing: left blue quadrant (downregulated genes) and right red quadrant (upregulated genes) relative to DMSO + HCMV. Right-shifted positions (positive values) and left-shifted positions (negative values) reflect increasing fold-change magnitude. Higher -log10(qval) values denote increased statistical significance of gene expression differences. (D) RNA-seq quantification of NR4A1 expression levels across four experimental groups. DMSO + Mock: WI-38 cells treated with vehicle control (0.02% DMSO) but not infected with HCMV. DMSO + HCMV: Cells infected with HCMV (MOI = 0.5) and treated with vehicle control. AKBA + Mock: Cells treated with 8 μM AKBA but not infected. AKBA + HCMV: Cells simultaneously infected with HCMV and treated with 8 μM AKBA. (E) Western blot analysis of NR4A1 protein levels in WI-38 cells infected HCMV (MOI = 0.5) and treated with 8 μM DMSO or AKBA, harvested at 3 days post-infection. (F) Quantification analysis of NR4A1 protein expression from three independent experiments. The vehicle control contained 0.02% (v/v) DMSO. *p < 0.05, **p < 0.01, ****p < 0.0001.

Comparative analysis of differentially expressed genes (DEGs) among the DMSO + Mock, DMSO + HCMV, and AKBA + HCMV groups. From the DEGs, we screened for the top 20 genes that met the following criteria: low expression in both DMSO + Mock and AKBA + HCMV groups, but high expression in the DMSO + HCMV group. A heatmap was generated using the OmicStudio advanced heatmap tool (https://www.omicstudio.cn/tool/4) (Fig. 4A). Among these candidates, NR4A1 emerged as a key factor, which is highly associated with herpesviruses. This finding aligns with recent evidence demonstrating NR4A1 as a critical host factor for herpesvirus propagation (Tommasi et al., 2020). This significant correlation motivated further investigation of NR4A1.

Volcano plot analysis was performed using the OmicStudio advanced tool (https://www.omicstudio.cn/tool/7) to generate volcano plots of DEGs, labeling the NR4A1 gene to visually assess its differential expression (upregulation or downregulation) across group comparisons. Comparison between DMSO + HCMV and DMSO + Mock groups revealed significant NR4A1 upregulated (log2FC = 2.12, qval < 0.001; Fig. 4B). Conversely, AKBA + HCMV treatment resulted in marked NR4A1 downregulation relative to DMSO + HCMV (log2FC = -3.19, qval < 0.001; Fig. 4C). NR4A1 Fragments Per Kilobase of transcript per Million (FPKM) mapped reads across all four groups were further illustrated in a bar chart (Fig. 4D).

Western blotting analysis at 3 dpi confirmed these findings, showing significantly reduced NR4A1 protein expression in AKBA + HCMV group compared with the DMSO + HCMV control (Fig. 4E and 4F). These findings indicate that AKBA exerts a significant inhibitory effect on HCMV-induced NR4A1 expression at both transcriptional and translational levels, suggesting that AKBA may inhibit HCMV infection via NR4A1.

### NR4A1 knockdown reduces HCMV infection in WI-38 cells

**Fig. 5.**
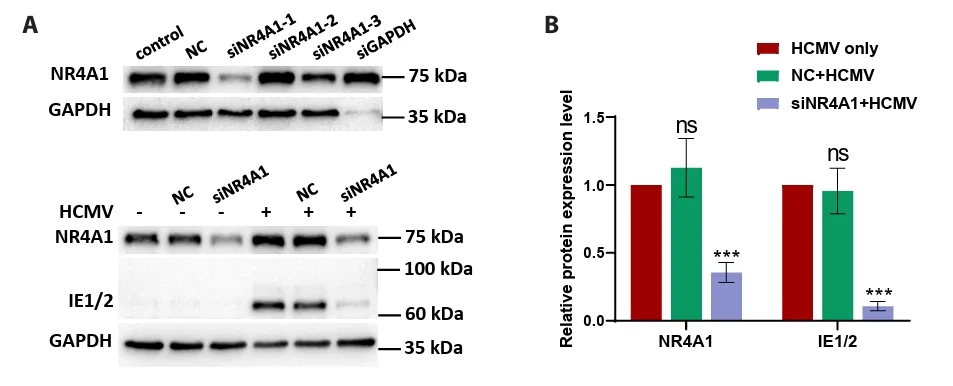
Knockdown of the host factor NR4A1 inhibits HCMV infection. (A) Screening for efficient NR4A1-targeting siRNAs. WI-38 cells were transfected with three independent NR4A1-targeting siRNAs (siNR4A1-1, siNR4A1-2, and siNR4A1-3), a non-targeting control siRNA (NC), or a positive control GAPDH siRNA (siGAPDH). Cells were harvested 72 h post-transfection, and NR4A1 protein levels were analyzed by Western blotting. siNR4A1-1 exhibited the highest knockdown efficiency and was selected for subsequent experiments. (B) Effect of NR4A1 knockdown on HCMV protein expression. WI-38 cells transfected with siNR4A1 or NC were infected with HCMV (MOI = 0.5). Cells were harvested 72 h post-infection, and protein levels of NR4A1 and the HCMV immediate-early protein IE1/2 were analyzed by Western blotting. GAPDH served as a loading control. (C) Quantification of NR4A1 and IE1/2 protein levels from B (HCMV only, NC + HCMV, and siNR4A1 + HCMV groups) using ImageJ. The data represent grayscale analysis from three independent experiments. ***p < 0.001; ns, not significant vs. HCMV only group.

To further validate the critical role of NR4A1 in HCMV infection, we performed siRNA-mediated gene knockdown experiments. By screening three independent NR4A1-targeting siRNAs, we first confirmed that siRNA transfection did not significantly affect WI-38 cell viability (Fig. S1). Subsequently, we found that siNR4A1-1 most effectively reduced NR4A1 protein expression in WI-38 cells, and this siRNA was selected for subsequent experiments (Fig. 5A). Subsequently, cells with NR4A1 knockdown were infected with HCMV (MOI = 0.5). The results showed that, compared to the HCMV only group, the expression of the viral protein IE1/2 was significantly reduced in the NR4A1-knockdown HCMV-infected group (Fig. 5B and 5C). These data confirm that NR4A1 is a host factor essential for efficient HCMV infection and support the hypothesis that AKBA exerts its antiviral activity by inhibiting NR4A1 expression (Fig. 4E and 4F).

## Discussion

HCMV infection poses significant clinical challenges in immunocompromised populations e.g., organ transplant recipients, AIDS patients, manifesting as pneumonia, retinitis, and multi-organ failure (Zhang et al., 2023). Current antiviral therapies, such as ganciclovir, are limited by adverse effects including myelosuppression and nephrotoxicity, as well as the emergence of drug-resistant strains (Senaweera et al., 2022; Wild et al., 2023). Natural products, with their favorable safety profiles and multi-target mechanisms, represent a promising source for novel antiviral discovery (Bradley et al., 2024; Lauß et al., 2024). This study provides the first systematic demonstration of the anti-HCMV activity of AKBA and elucidates its underlying molecular mechanism.

Our results demonstrate that AKBA, within a non-cytotoxic concentration range (Fig. 1), dose-dependently and significantly inhibits HCMV replication in WI-38 cells. This inhibition was evidenced by reduced cytopathic effect (CPE), downregulated expression of viral proteins (IE1/2, p52), decreased viral gene copy numbers (*UL123*, *UL44*, and *UL32*), and a dose-dependent decline in infectious viral progeny titer (TCID_50_) (Fig. 2). Subsequent time-of-addition experiments elucidated that AKBA primarily interferes with the viral lifecycle during the post-entry phase within host cells, while it also can directly inactivate free viral particles extracellularly (Fig. 3). To dissect the mechanism of AKBA's action in the post-entry stage, we performed transcriptomic analysis and identified the host factor NR4A1 as a key element that is markedly upregulated upon HCMV infection and whose expression is reversed by AKBA treatment (Fig. 4). Subsequent knockdown experiments confirmed that NR4A1 is an essential host factor for efficient HCMV replication (Fig. 5). Therefore, this study not only confirms that AKBA potently and dose-dependently suppresses HCMV replication in WI-38 cells, but also reveals the mechanisms underlying its antiviral activity: the downregulation of the critical host factor NR4A1 and the direct inactivation of HCMV particles.

The viral lifecycle encompasses multiple key stages, including attachment, entry, gene expression, replication, assembly, and release (Levrier et al., 2025). Therefore, identifying the specific stage at which a drug exerts its effect is crucial for antiviral research. Time-of-addition experiments elucidated two distinct stages of AKBA's anti-HCMV action: it not only effectively inhibits viral replication during the post-entry stage but also directly inactivates free viral particles. AKBA demonstrated the most potent inhibitory effect when administered after viral entry (Post-T) (Fig. 3B and 3C), clearly indicating that its primary target lies within the intracellular processes following viral entry into the host cell. In this respect, AKBA exhibits a similarity to first-line anti-HCMV drugs such as ganciclovir (Zhang et al., 2021). Notably, this study also revealed that AKBA possesses direct virucidal activity, as evidenced by the loss of viral infectivity after in vitro pre-incubation (Pre virus & drug-T) (Fig. 3B, 3C, 3E, and 3F). Direct inactivation of free viral particles can rapidly reduce the extracellular viral load, potentially limiting cell-to-cell spread (Shirvanimoghaddam et al., 2021). This suggests that AKBA merits exploration for its potential application as a topical formulation.

NR4A1 (Nuclear Receptor Subfamily 4 Group A Member 1) is an orphan nuclear receptor transcription factor that broadly regulates cellular physiological and pathological processes (Safe et al., 2021). Previous studies have shown that in varicella-zoster virus (VZV) infection, which also belongs to the *Herpesviridae* family, NR4A1 promotes viral assembly and maturation by modulating cellular autophagy pathways (Tommasi et al., 2020). We speculate that in HCMV infection, NR4A1 may employ similar mechanisms. Specifically, it might facilitate the establishment of an intracellular environment conducive to viral replication by recruiting epigenetic modifier complexes to regulate viral gene transcription (Liu et al., 2024; Wan et al., 2023), or by suppressing type I interferon signaling to dampen the host innate immune response (Chen et al., 2025; Yao et al., 2023). The downregulation of NR4A1 by AKBA could potentially disrupt these host processes hijacked by the virus.

The potent anti-HCMV activity of AKBA revealed in this study highlights its potential as an antiviral compound. The pharmacological profile of AKBA in terms of cytotoxicity aligns with features commonly observed in many natural products during early development. Future research could focus on exploring structural modifications and advanced formulations, such as nano-delivery systems, to improve its selectivity index, strategies that have shown promise for enhancing the selectivity index of other triterpenoids in prior studies (He et al., 2025).

## Figures and Tables

**Table 1.** List of primers

Primer name	Primer sequence (5'-3')
*UL123*-F	TCTGCCAGGACATCTTTCTC
*UL123*-R	GTGACCAAGGCCACGACGTT
*UL44*-F	ACTGCCGTGCACGTTGCGTA
*UL44*-R	ACTTGCCGCTGTTCCCGACG
*UL32*-F	GGTTTCTGGCTCGTGGATGTCG
*UL32*-R	CACACAACACCGTCGTCCGATTAC
*GAPDH*-F	CTGTTGCTGTAGCCAAATTCGT
*GAPDH*-R	ACCCACTCCTCCACCTTTGAC

**Table 2.** List of siRNA

siRNA	siRNA sequence (5'-3')
si*NR4A1*-1-Sense	GCACCUUCAUGGACGGCUA dTdT
si*NR4A1*-1-Antisense	UAGCCGUCCAUGAAGGUGC dTdT
si*NR4A1*-2-Sense	CCUUCAAGUUCGAGGACUU dTdT
si*NR4A1*-2-Antisense	AAGUCCUCGAACUUGAAGG dTdT
si*NR4A1*-3-Sense	UGGUGAAGGAAGUUGUCCGAA dTdT
si*NR4A1*-3-Antisense	UUCGGACAACUUCCUUCACCA dTdT

## Supplementary Information

Fig. S1. Effect of siRNA transfection on the viability of WI-38 cells. WI-38 cells were transfected with non-targeting control siRNA (NC), three independent NR4A1-targeting siRNAs (siNR4A1-1, siNR4A1-2, and siNR4A1-3), or a positive control GAPDH siRNA (siGAPDH). At 48 h post-transfection, relative cell viability was measured using the CCK-8 assay. Data are presented as Mean ± SD (n = 3). Statistical significance was determined by one-way ANOVA compared to the Control group; ns indicates no significant difference.
