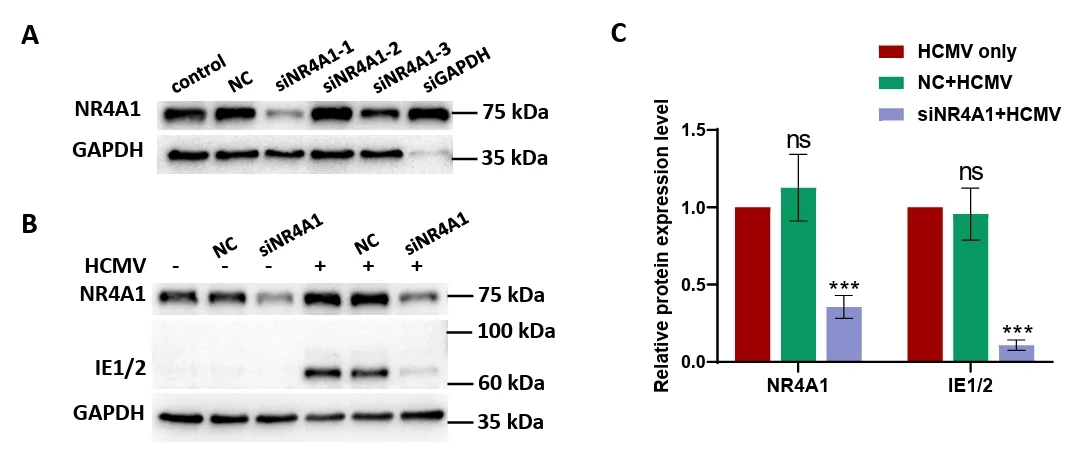# Erratum: Inhibitory effects of Acetyl-11-keto-β-boswellic acid (AKBA) on human cytomegalovirus (HCMV) in vitro

**DOI:** 10.71150/jm.2607100

**Published:** 2026-07-31

**Authors:** Bingquan Chu, Zhiwei Ding, Xinna Wu, Yunchuang Chang, Chunxia Wu, Yicheng Fu, Genxiang Mao, Sanying Wang

**Affiliations:** 1 School of Biological and Chemical Engineering, Zhejiang University of Science and Technology, Hangzhou 310023, P. R. China; 2 Zhejiang Key Laboratory of Geriatrics and Geriatrics Institute of Zhejiang Province, Zhejiang Hospital, Hangzhou 310030, P. R. China

Erratum: Journal of Microbiology. 2026. 64: e2601007

https://doi.org/10.71150/jm.2601007

­

The original online version of this article was revised:

This article contained an error in the panel labels of Fig. 5. The labels have been corrected from A and B to A, B, and C. This correction does not affect the results, interpretation, or conclusions of the article.

## Results

### Published Fig. 5

The panel labels were incorrectly presented as A and B.

[Fig f1-jm-2607100]

### Corrected Fig. 5

The panel labels have been corrected to A, B, and C.

[Fig f2-jm-2607100]

## Figures and Tables

**Figure f1-jm-2607100:**
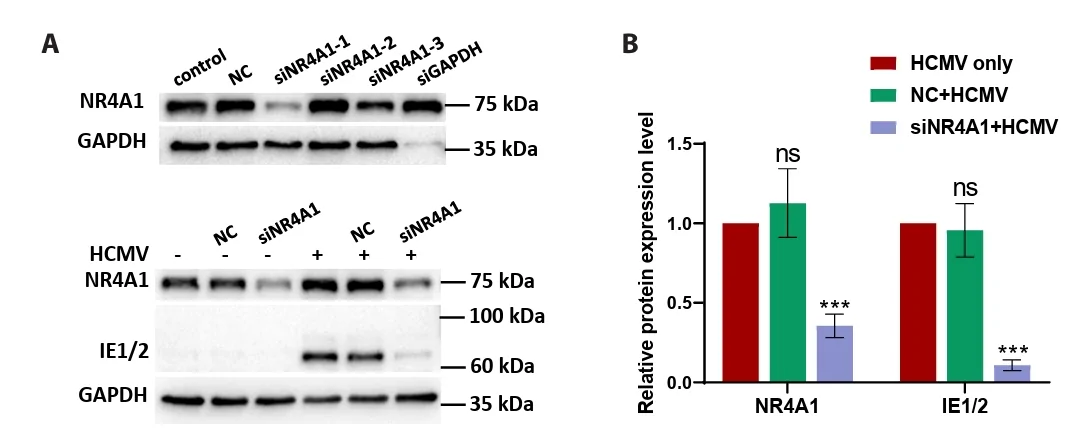


**Figure f2-jm-2607100:**