# Supplementary material for: Inhibitory effects of acetyl-11-keto-β-boswellic acid (AKBA) on human cytomegalovirus (HCMV) in vitro
Source: J Microbiol. 2026 Mar 25;64(4):e2601007. doi: 10.71150/jm.2601007 (PMC13577096; doi:10.71150/jm.2601007)
Supplement: Supplementary file 1 [file jm-2601007-Supplementary-Fig-S1.pdf]

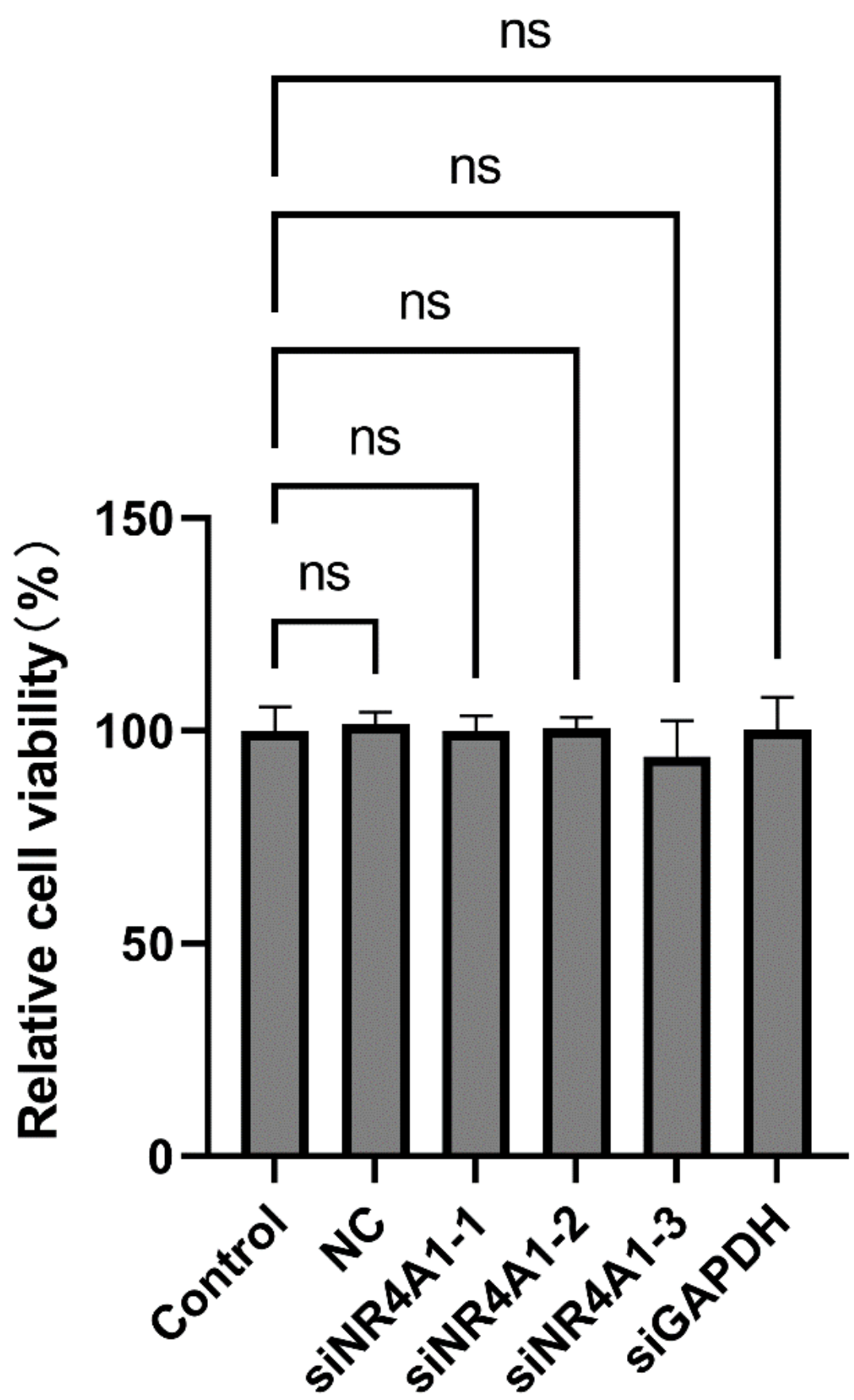

Fig. S1. Effect of siRNA transfection on the viability of WI-38 cells. WI-38 cells were transfected with non-targeting control siRNA (NC), three independent NR4A1-targeting siRNAs (siNR4A1-1, siNR4A1-2, and siNR4A1-3), or a positive control GAPDH siRNA (siGAPDH). At 48 h post-transfection, relative cell viability was measured using the CCK-8 assay. Data are presented as Mean ± SD (n = 3). Statistical significance was determined by one-way ANOVA compared to the Control group; ns indicates no significant difference.
